# One academic year under COVID-19 conditions: two multicenter cross-sectional evaluation studies among medical students in Bavarian medical schools, Germany students’ needs, difficulties, and concerns about digital teaching and learning

**DOI:** 10.1186/s12909-022-03480-x

**Published:** 2022-06-10

**Authors:** Christopher Holzmann-Littig, Nina L. Zerban, Clara Storm, Lilian Ulhaas, Mona Pfeiffer, Alexander Kotz, Marjo Wijnen-Meijer, Stephanie Keil, Johanna Huber

**Affiliations:** 1 TUM Medical Education Center, Faculty of Medicine, Technical University of Munich, Munich, Germany; 2 Department of Nephrology, University Hospital rechts der Isar, Faculty of Medicine, Technical University of Munich, Munich, Germany; 3 Institute of Medical Teaching and Medical Education Research, University Hospital of Würzburg, Würzburg, Germany; 4 Simulated Patient Program, Faculty of Medicine, Julius-Maximilians-Universität of Würzburg, Würzburg, Germany; 5 Institute for Medical Education, University Hospital, Ludwig-Maximilians-Universität Munich, Munich, Germany; 6 Faculty of Medicine, Dean’s Office and Department of Medical Education Augsburg DEMEDA, Augsburg University, Augsburg, Germany; 7 Faculty of Medicine, Office of the Dean for Student Affairs, Friedrich-Alexander-Universität Erlangen-Nürnberg, Erlangen, Germany; 8 Faculty of Medicine, Dean’s Office, University of Regensburg, Regensburg, Germany

**Keywords:** COVID-19, Pandemics, Medical education, Medical students, Medical schools, Distance education, Digital teaching and learning, New Normal, Evaluation study

## Abstract

**Background:**

Since March 2020, COVID-19 has created a need for adaptation in many areas of life. This study explores medical students’ perspectives on digital teaching under conditions of the COVID-19 pandemic. It focuses on expectations and concerns about digital teaching, the evaluation of specific aspects of teaching, and requests for future teaching.

**Methods:**

Six German faculties have joined forces within the Bavarian network for medical education to develop and deploy a common core questionnaire. Cross-sectional surveys were conducted at the end of the summer semester 2020 and winter semester 2020/21. Medical students from different semesters participated in the online survey. Data was analyzed descriptively and/or inferentially. Item differences across semesters were examined using contingency tables and Chi^2^ tests. Mean values were compared using the independent samples t-test; answer frequencies in retrospective and prospective concerns were compared using contingency tables and Chi^2^ tests with Yates’ correction.

**Results:**

In the summer semester 2020, 1565 students and in winter semester 2020/21, 1727 students took part in the survey. Students’ main prospective concern was lack of social exchange between fellow students (70%), but also with teachers. Second and third most often concerns were a lack of practical training (68%) and lack of integration of on-site digital teaching (50%). Approximately 7% of the students lacked sufficient access to technical equipment.. Approximately 39% of the students lacked a sufficient internet connection for synchronous digital teaching, 17% for asynchronous digital teaching. On-site teaching was the preferred form of teaching (60%), and there was a preference for asynchronous (24%) over synchronous (15%) digital teaching. Teaching recordings (79%) were particularly popular to complement future on-site teaching.

**Conclusions:**

The following areas of education under COVID-19 conditions are highly important to medical students: adequacy of information sharing, integration of opportunities for exchange with fellow students and teachers, possibility to perform practical trainings. After the normalization of the pandemic situation, on-site teaching should be supplemented with blended learning concepts such as the inverted classroom model.

**Graphical abstract:**

Percentages of results are rounded averages from summer and winter semesters.
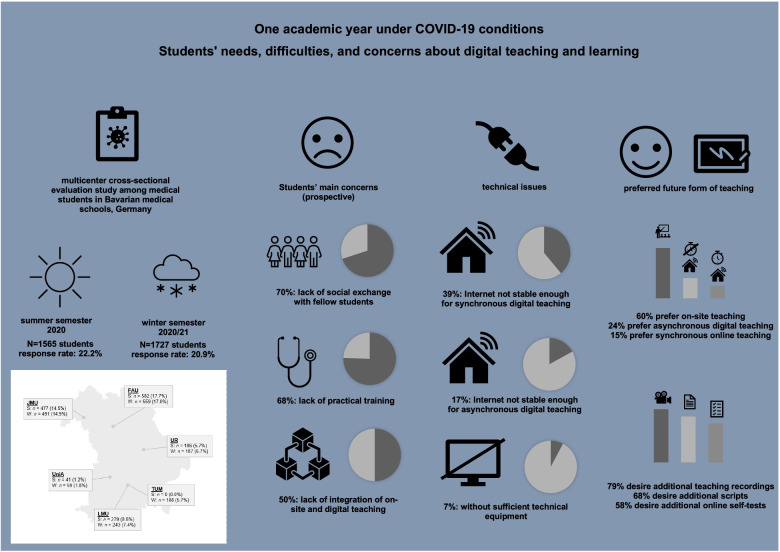

**Supplementary Information:**

The online version contains supplementary material available at 10.1186/s12909-022-03480-x.

## Background

With the emergence of the Coronavirus SARS-CoV-2 (COVID-19) pandemic and the associated government-imposed public health measures (e.g. comprehensive hygiene measures, physical distancing, quarantine, travel restrictions) [1] as well as social and economic and political burdens [2, 3], universities around the world were forced to switch in a rush from predominantly on-site teaching to digital teaching at the beginning of the summer semester 2020 [4].

Medical schools in Germany and around the world had to adapt their curricula to the new situation as quickly as possible [5–9]. Some of them were able to draw on experience and existing concepts in digital teaching, such as blended learning and the inverted classroom method [10–12]. Others could only refer to little or no experience in digital teaching [13]. Nevertheless, medical schools around the world had one thing in common: all available resources had to be mobilized to set up an emergency remote teaching with the help of practical and creative solutions [14].

According to systematic reviews, digital medical teaching can be effective [15]; useful approaches include telehealth, social media and video conferencing [16]. Nonetheless, digital approaches are not equally well suited to all teaching formats, and particular difficulties arise in implementing hands-on, patient-centered teaching [17].

Parallel to the conversion of teaching and testing, medical schools developed concepts for the evaluation of digital teaching. On the one hand, to be able to ensure the quality of teaching despite this exceptional situation. On the other hand, to be able to answer, among others, the following questions [5, 7, 11, 13, 18–27]:

- How is digital teaching designed within the curriculum? Which concepts, methods, tools, digital platforms and software programs are used to teach content?
- What do students and teachers particularly like about digital teaching?
- What are conducive or hindering factors with regard to the implementation of digital teaching concepts and the participation of students in digital teaching?
- Which digital teaching concepts should be maintained beyond the COVID-19 pandemic and how can they be implemented in the curricula in a didactically valuable way?

Some medical schools have also investigated the cognitive, emotional and psychological effects of the COVID-19 pandemic and the resulting digital teaching on their students [6, 28–30]. Medical students have been and continue to be on the front lines of pandemic response. For this, structured preparation, such as that provided by the sample course by Ashcroft and colleagues, is essential [31], but there must also be clearly delineated role definitions [32].

According to a Best Evidence Medical Education (BEME) scoping review, previous publications on medical teaching under COVID-19 conditions focused primarily on the issues of simulation and distance teaching and provided evidence of the importance of collaboration [33]. Around the world, medical schools have undertaken extensive evaluation efforts [11, 13, 28, 29]. Some of them have developed a regional or even national strategy for evaluating digital teaching [6, 7, 12, 34, 35]. In Germany, national institutions for higher education development, such as the Centre for Higher Education (Centrum für Hochschulentwicklung; CHE), the German Centre for Higher Education Research and Science Studies (Deutsches Zentrum für Hochschul- und Wissenschaftsforschung; DZHW) or the Institute for Higher Education Development (Institut für Hochschulentwicklung; HIS), have conducted national surveys on the quality and future of digital teaching at German universities [4, 10, 36, 37]. However, evaluation data on digital teaching at medical schools based on jointly developed questionnaires is scarce.

For this reason, medical faculties have joined forces within the Competence Network Medical Education in Bavaria (Kompetenznetz Medizinlehre Bayern; KMB) to be able to generate cross-site evaluation results and conclusions for digital teaching. Our research questions were:

1. How do medical students in Bavaria perceive the ad hoc conversion to pure digital teaching? What are their concerns and expectations?
2. Which aspects of digital teaching do medical students in Bavaria consider to be particularly successful? What would they like to maintain in the future?
3. Which aspects need to be improved with regard to future digital teaching?

## Methods

We conducted two cross-sectional surveys [38] among medical students in Bavaria (Germany) at six medical schools: Universität Augsburg (UniA); Friedrich-Alexander-Universität Erlangen-Nürnberg (FAU); Ludwig-Maximilians-Universität München (LMU); Technische Universität München (TUM); Universität Regensburg (UR); Julius-Maximilians-Universität Würzburg (JMU). The aim was to investigate students’ assessment of medical education under COVID-19 conditions. This study refers to the summer semester 2020 (courses taught from April to July 2020; this was the first semester affected by COVID-19) and winter semester 2020/21 (courses taught from October 2020 to February 2021). The survey waves took place from July 2020 to October 2020 and from January 2021 to April 2021.

In total, 7053 students in the summer semester 2020 and 8279 students in winter semester 2020/21 were asked to participate in the study. All students participated voluntarily and completely anonymously. We sent invitations by E-mail at all sites and conducted the survey using the evaluation software *EvaSys* (*evasys GmbH*) as part of the respective semester evaluation.

At FAU^1^ and JMU, all human medicine students were surveyed. LMU and UR chose to survey only students in the clinical phase of their studies. At UniA, the human medicine program had only begun in the winter semester 2019/20, so it was not possible to survey students in higher semesters there. For the summer semester, no data is available from TUM, as this site had already conducted an independent survey in advance. In the winter semester, students from the clinical phase took part in the survey as the pre-clinical phase is being held at LMU for students from TUM and LMU together.

The core questionnaire contains 29 questions in total (20 closed and 9 open ones). In this study, we focus on the closed questions. Questions with related content were combined into one question group. The GESIS survey guidelines [39] for question formulation were considered in question construction. In total, the questionnaire consists of five question groups:

- Organizational framework
- Technological framework
- Communication and interaction
- Online teaching
- Overall assessment

For questions on the functionality of the personal technical equipment and the internet connection, the answer options “Yes” / “Partially” / “No” were formed. For questions on preferred means of communication, time of day for working on learning materials, online activities, and expressed concerns the respective options were offered for selection (“selected” / “not selected”). If “other” was selected, free text entry was allowed. The offered teaching forms could be prioritized in descending order. For questions involving a strength of agreement, 5-point Likert scales were formed. The five-point Likert scales had the endpoints “fully applies” and “does not apply at all”. The five-point Likert scale for the item “The flexible time management makes working on the learning units” had the endpoints “much easier” and “much more difficult”. In the overall evaluation of the semester, the maximum scores were “very good” and “poor”. The complete questionnaire is available in German (original) and an English version (translation) in Supplement 1. The terms “online teaching” and “digital teaching” were used synonymously in the questionnaire.

A detailed description of the survey procedure can be found in Supplement Table 2. The CHERRIES checklist for reporting online surveys [40] was applied for this purpose. Incomplete questionnaires were included in the analysis, the percentages are corrected for the respective number of responding students.

**Fig. 1:**
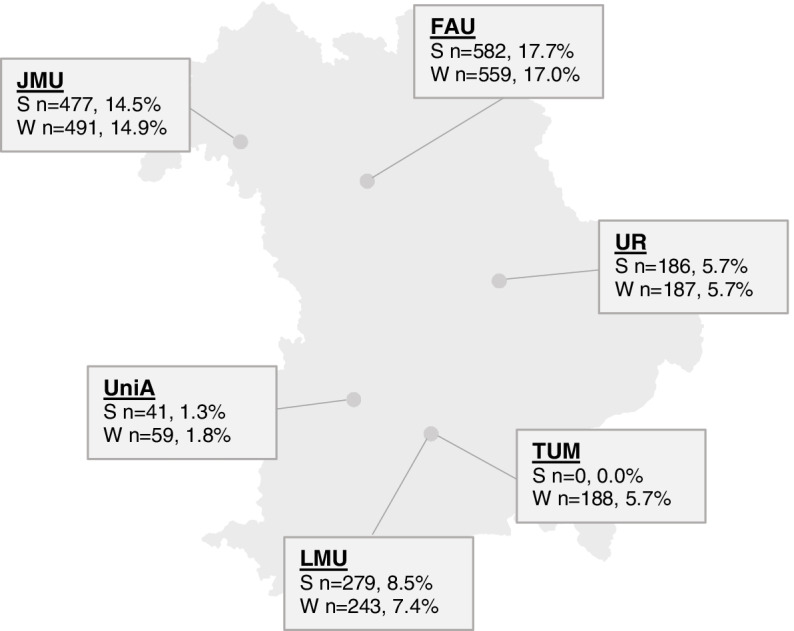
Number of data sets per site. Number of data sets per site. Total participants: 3292. *n* = number of participants per site, % = percent of total participants across both semesters; S = summer semester 2020, W = winter semester 2020/21, JMU = Julius-Maximilians-Universität Würzburg, FAU = Friedrich-Alexander-Universität Erlangen-Nürnberg, UR = Universität Regensburg, UniA = Universität Augsburg, TUM = Technische Universität München, LMU = Ludwig-Maximilians-Universität München. Percentages do not add up to 100% due to rounding

Statistical analysis was performed using JASP, version 0.14.1 [41]. Descriptive results were reported as frequencies, mean values and standard deviation, or minimum and maximum. Contingency tables were formed and Chi^2^ tests were performed to determine differences between items in the summer and winter semester groups. Mean value comparisons were performed using the independent samples t-test. Comparisons of answer frequencies in retrospective and prospective concerns were performed with contingency tables, using a Chi^2^ test with Yates’ correction in the GraphPad calculator [42]. The template for Fig. 1 was created in R (version 4.0.4) with the package raster [43]. Bar charts were created using Microsoft Excel. A *p*-value of 0.05 was considered statistically significant.

## Results

The main findings from the study are highlighted below. Quantitative results for all questions asked in the core questionnaire can be found in Supplement Table 3.

### Participants

Over both survey waves, a total of 3292 data sets could be evaluated, which corresponds to a response rate of 22.2% (summer semester) and 20.9% (winter semester) respectively. The number of data sets per site can be seen in Fig. 1.

### Organizational framework

In the summer semester, students (*n* = 1500) agreed with an average of 3.7 (*SD* = 1.1) with the statement that they knew where to find information on the module^2^ and/or semester schedule – in the winter semester (*n* = 1695) this agreement was slightly stronger with an average of 4.0 (*SD* = 1.0); *p* ≤ 0.001.

Students considered the information provided also nearly as helpful in the summer semester (*n* = 1496, *M* = 3.8*, SD =* 0.9) as in the winter semester (*n* = 1689, *M* = 3.9, *SD* = 0.9); *p* ≤ 0.001.

However, students in the winter semester reported knowing a little more clearly whom to contact with questions (summer semester: *n* = 1485, *M* = *3.6, SD* = 1.2; winter semester: *n* = 1674, *M* = 3.8, *SD* = 1.2; *p* ≤ 0.001).

### Technological framework

Students most commonly used laptops both in the summer semester (85.5%, *n* = 1303) and the winter semester (86.4%, *n* = 1492). Tablets were used the second most in both semesters (summer: 42.2%, *n* = 643; winter: 41.2%, *n* = 712). Smartphones played a minor role for studying with 15.8% (*n* = 241) mentions in the summer semester and 11.1% (*n* = 192) in the winter semester. The decrease in smartphone use was statistically significant (*p* ≤ 0.001). In the summer semester, 10.6% (*n* = 162) of the responding students primarily used a desktop PC for learning; this tendency was stable in the winter semester at 12.6% (*n* = 217).

In the summer semester, 92.5% (*n* = 1389) of students reported being able to participate in synchronous online sessions with their technical equipment; this proportion was similar in the winter semester with 93.7% (*n* = 1594).

A webcam was available to 90.7% (*n* = 1361) of the participants in the summer semester and 92.7% (*n* = 1570) of the participants in the winter semester; *p* = 0.002.

In the summer semester, 83.0% (*n* = 1247) of students reported that their internet connection was stable enough to view instructional videos; in the winter semester, this was the case for 83.5% (*n* = 1419).

However, only 62.6% (*n* = 940) of the participants in the summer semester and 60.6% (*n* = 1030) of the participants in the winter semester considered the internet connection stable enough for participation in interactive, synchronous online sessions.

### Communication with teachers

In both semesters, students agreed with the statement about missing the personal contact with teachers. However, this agreement was more pronounced in the winter semester (summer semester: *n* = 1539, *M* = *3.6, SD = 1.2*; winter semester: *n* = 1690, *M* = *3.8, SD* = 1.2; *p* ≤ 0.001).

**Fig. 2:**
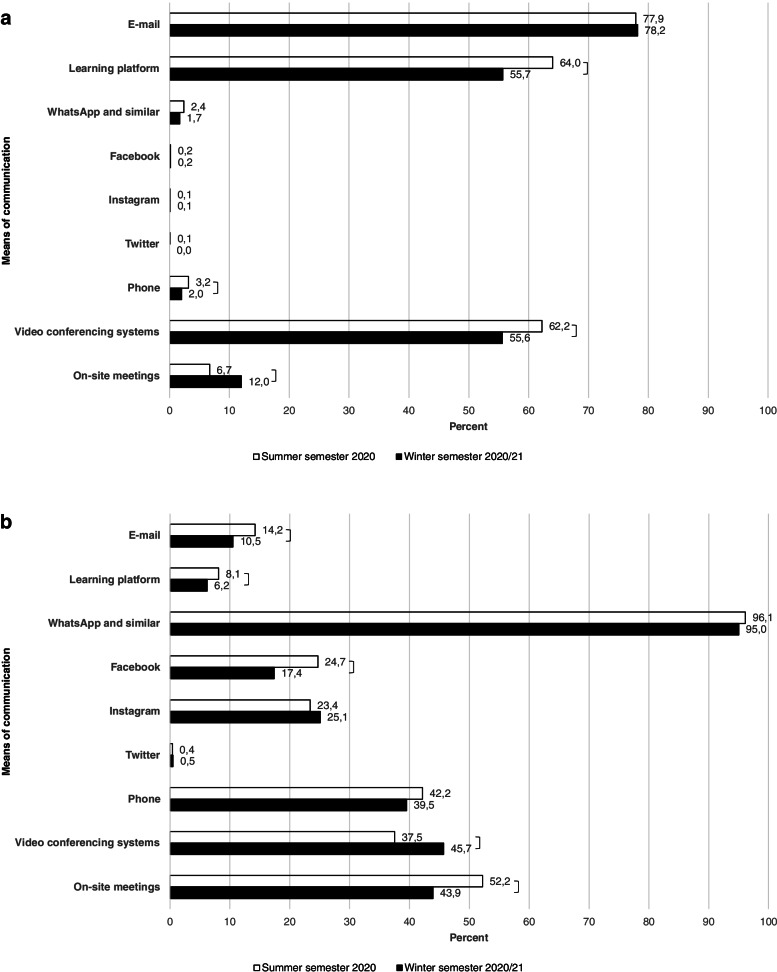
**a** Means of communication used between students and teachers. *Notes*: summer semester 2020: *n* = 1524, winter semester 2020/21: *n* = 1727; multiple selection possible; * *p* ≤ 0.05; *** *p* ≤ 0.001. **b** Means of communication used between fellow students. *Notes*: summer semester 2020: *n* = 1524, winter semester 2020/21: *n* = 1727; multiple selection possible; * *p* ≤ 0.05; *** *p* ≤ 0.001

Students primarily held contact with the faculty via E-mail in both semesters. Learning platforms were used second most often for this purpose. Video conferencing systems were used third most often. On-site meetings, phone, WhatsApp or similar, Facebook, Instagram and Twitter played a minor role in communication. The means of communication used between students and teachers are shown in Fig. 2a.

### Communication with fellow students

In the summer semester (*n* = 1543), students agreed with the statement that they missed the personal contact with fellow students with an average of 4.4 (*SD* = 1.0); in the winter semester (*n* = 1692) they agreed even more strongly, on average with 4.6 (*SD* = 0.9); *p* ≤ 0.001.

In the communication between fellow students, WhatsApp or similar appeared to be most important. This was followed by on-site meetings, phone calls, video conferencing, Instagram and Facebook. E-mails, learning platforms and Twitter played minor roles. The tools used in communication among fellow students are shown in Fig. 2b.

### Comparison with the previous semester

All in all, students neither agreed nor disagreed that the current semester was much more exhausting than the previous one (summer semester *n* = 1408, *M* = 3.1, *SD* = 1.4; winter semester *n* = 1444, *M* = 3.1, *SD* = 1.4); *p* = 0.675.

### Prioritization of teaching forms

When asked about their preferred way of teaching, on-site teaching ranked number one by 55.5% (*n* = 767) in the summer semester and 65.7% (*n* = 1031) in the winter semester. Asynchronous digital teaching was second most often ranked number one (summer semester: 27.4%, *n* = 378; winter semester: 21.0%, *n* = 329). Synchronous online teaching was the least often prioritized form of teaching (summer semester: 17.1%, *n* = 236; winter semester: 13.3%, *n* = 209).

### Preferred future form of teaching

When asked about online activities that should be kept for the future to supplement traditional on-site teaching, students most often selected teaching recordings. Scripts, etc. (e.g. slides, summary, journal article) were chosen second most often. Online self-tests were requested by more than half of the students in both the summer and winter semester.

**Fig. 3:**
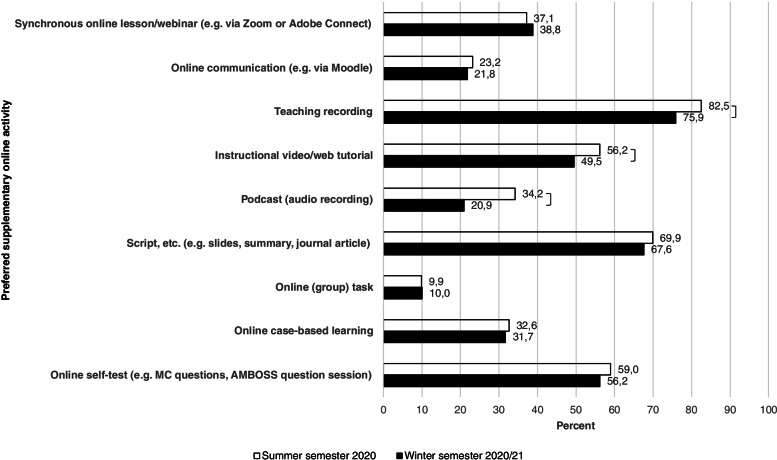
Evaluation of online activities to supplement traditional on-site teaching for future semesters. *Notes*: multiple selection possible; *** *p* ≤ 0.001

The evaluation of different online activities to complement traditional on-site teaching in the future can be seen in Fig. 3.

### Students’ concerns

In both survey waves, students were asked retrospectively about their concerns at the beginning of the respective semester and asked prospectively about their concerns regarding the upcoming semester.

Regarding the beginning of the summer semester 2020, students’ most often reported concern (78.8%) was not being able to perform practical trainings. This was reported less often (66.7%) regarding the upcoming winter semester 2020/21. In the second survey round, 80.0% of the students expressed this concern retrospectively for the beginning of the winter semester 2020/21 and 68.8% prospectively regarding the upcoming summer semester 2021.

The second strongest concern (78.3%) at the beginning of the summer semester 2020 was a lack of social exchange with fellow students. This significantly decreased to 64.2% regarding the upcoming winter semester 2020/21. In the second survey round, this concern was reported most often with 84.3% for the beginning of the winter semester 2020/21 and was still present at a high level with 75.9% regarding the upcoming summer semester 2021.

The third strongest concern regarding the beginning of the summer semester 2020 was poor information about the organization from the part of the faculty with 71.2%. This concern was expressed by 37.6% regarding the upcoming winter semester 2020/21. In the second survey round, it was at 55.4% regarding the beginning of the winter semester 2020/21 and at 31.4% regarding the upcoming summer semester 2021.

Concerns regarding the personal digital knowledge or technical equipment as well as concerns regarding the ability to use the learning platform were much less pronounced among students. The item regarding concerns about an insufficient integration of on-site and digital teaching was only asked in relation to the following semester.

**Fig. 4:**
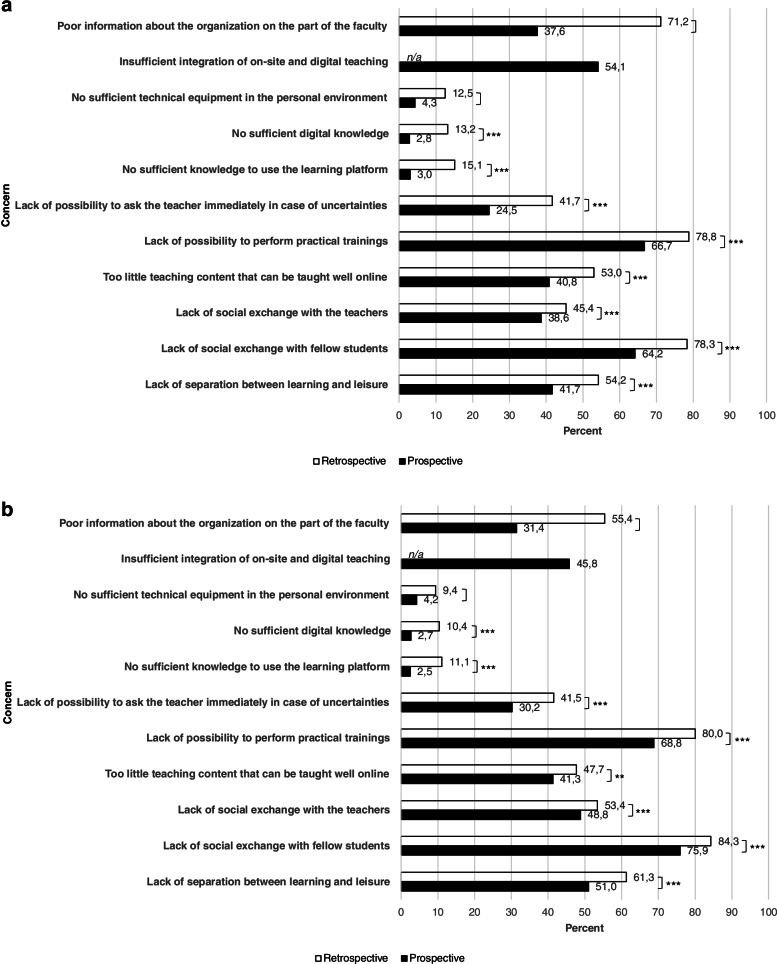
**a** Student concerns as surveyed at the end of the summer semester 2020. *Notes*: *n* = 1524; retrospective = concerning the beginning of the summer semester 2020; prospective = concerning the upcoming winter semester 2020/21; multiple selection possible; *** *p* ≤ 0.001. **b** Student concerns as surveyed at the end of the winter semester 2020/21. *Notes*: *n* = 1727; retrospective = concerning the beginning of the winter semester 2020/21; prospective = concerning the upcoming summer semester 2021; multiple selection possible; ** *p* ≤ 0.01; *** *p* ≤ 0.001

Figure 4a shows retrospective and prospective student concerns as surveyed at the end of the summer semester 2020. Figure 4b shows retrospective and prospective student concerns as surveyed at the end of the winter semester 2020/21.

## Discussion

In summary, this study exposes the following areas for further development of medical teaching under COVID-19 conditions at Bavarian faculties: 1) offering transparent and well-structured information, 2) ensuring access to synchronous online teaching, and 3) providing opportunities for social interaction with fellow students. It also reveals students’ general preference for on-site teaching and their choice of asynchronous digital teaching over synchronous online teaching. There was a particular desire for the future use of teaching recordings to supplement on-site teaching. The most common concerns of students were the lack of possibility to perform practical trainings / lack of integration of digital and on-site teaching and the lack of social exchange with fellow students.

At this point, we would like to reference to materials that we would have liked to have at hand some earlier. When having to choose between various methods in medical education under pandemic conditions, Lim and colleagues’ decision tree can be helpful [44]. For the implementation of digital medical education under COVID-19, we recommend the 12 practical tips by Jiang and colleagues [45]. A comparison between different digital communication tools and didactic approaches of several medical schools can be found in an article by Chatterjee and Chakraborty [46]. Recommendations for pandemic-compliant medical skills practice can be found in an article by Hall and colleagues [47]. Those involved in developing and administering student exams will find relevant assessment forms in Gupta and colleagues’ overview, which also sheds light on the respective advantages and disadvantages in the context of the pandemic [48].

Our results on the organizational framework indicate that the Bavarian medical faculties, despite the challenging and constantly changing COVID-19 situation, have improved their information management in the course of the pandemic. The administrations of the medical faculties seem to be able to fall back on a certain resilience in their structures in this regard [49]. However, the “need for effective crisis communication practices” discussed by Su et al. in relation to the media [50] certainly also applies to universities / medical schools. Further research on effective, rapid, clear, and safe crisis communication with students therefore appears to be needed. The communication channels preferred by students in our study may not be fully generalizable due to regional differences. For example, a study from Pakistan showed a significantly lower proportion of WhatsApp / Facebook use than among our students [51]. It may therefore be potentially helpful for medical schools to consider the communication channels established among their students when planning their communication with students for possible further crisis situations.

In addition, the student evaluations of the organizational framework, which are in the upper middle range, indicate that the Bavarian medical faculties still have a need for development with regard to a more transparent and better-structured information policy. However, since we do not have comparative values on the organizational framework before COVID-19, it is difficult to assess how great the need for development of the administration actually is.

Even after the second COVID-19 semester, the data reveals that in terms of students’ technical equipment and access to a stable internet connection, a non-negligible proportion of students is unable to participate in synchronous online sessions, or only to a limited extent. These students are at risk of being left behind — universities should find ways to compensate for social disadvantages in the use of digital media, otherwise worse-off students will be at an even greater disadvantage in the future than this is already the case [19]. The provision of teaching recordings of synchronous online sessions, pandemic-friendly workspaces with a stable internet connection and the expansion of university rental services for technical equipment such as laptops could have a cushioning effect.

Students especially missed the personal contact with fellow students, but also with teachers. In the second COVID-19 semester this intensified further. Other surveys already reported that physical distancing and the shift to digital teaching decreased communication between students as well as between students and faculty in comparison to teaching before the pandemic [13, 19, 36]. It can be concluded that universities should find ways to minimize the social isolation of their students when relying more on e-learning concepts in the future. While students kept in touch with each other mainly via WhatsApp and comparable instant messaging services, e-mails were the most important communication channel between students and teachers. In phases in which e-learning must be used primarily, it may be worth offering communication opportunities via video conferencing systems, to allow for somewhat more personal interaction. In the winter semester, presumably after working out suitable hygiene concepts, teachers were again able to offer more on-site meetings than in the summer semester. On-site meetings among students, on the other hand, decreased in the winter semester, which can be explained by contact restrictions in Bavaria. Over the course of the pandemic, students increasingly turned to video conferencing for peer-to-peer communication, which can also be seen as a good preparation for future online consultations.

Students did not evaluate the summer semester (first semester under COVID-19 conditions) as more exhausting than the previous semester with traditional on-site teaching. Similar, this is the case for the comparison between the two semesters under COVID-19 conditions: The winter semester 2020/21 was not rated as more exhausting than the preceding summer semester. Publications with a mental health focus nevertheless suggest pressures from studying in times of COVID-19 that should not be underestimated [29, 30].

The most frequently preferred form of teaching was on-site teaching, which probably reflects the desire for personal contact. The fact that asynchronous digital teaching was preferred over synchronous online teaching could be explained by the desire for temporal flexibility, which is especially critical for student crisis workers. Rahmat et al. also found “a positive end explicit approach to” using e-library services among medical students, which are also an asynchronous and flexible form of e-learning [52]. While the preference for on-site teaching increased in the winter semester, the preference for digital teaching formats decreased. This could be due to the permanent burden of the pandemic and “webinar fatigue” [53].

The most popular online activity to supplement future on-site sessions is teaching recordings, although these declined in popularity somewhat in the winter semester. Scripts, etc. (e.g. slides, summary, journal article) ranked second, and online self-tests ranked third. These frequently chosen online activities allow students flexibility in time and location, as well as the opportunity to study at their own pace. This explanation is in line with results of Dost and colleagues [7]. In addition, online self-tests can be used to check one’s own knowledge level and receive (automated) feedback. In their scoping review, Katz and Nandi point to the largely untapped potential of social media in medical education [54]. However, there was no checkbox to select this in the questionnaire used.

The biggest concern of students during the summer semester was the lack of possibility to perform practical trainings. Depending on the pandemic situation, practical training in small groups with a strict hygiene concept or creative digital substitutes can provide a remedy. During the winter semester, the biggest concern of students was the lack of social exchange with fellow students. At another German university (Universität Hamburg), the loss of direct interaction was also seen as a major cause for concern by students [55]. Working with breakout rooms within video conferences is one approach to providing students with a platform for social exchange. However, in the event of further pandemic waves, medical schools should also focus more on the mental health of their students and try to provide appropriate support services [56]. Another result of this study was that retrospectively reported concerns related to the beginning of the semester were always higher than prospective concerns related to the following semester. At this point, the retrospective assessment method of concerns could be criticized. However, when interpreting the differences between semesters in terms of content, the experience made during the respective semester may have reassured students about the following semester.

In their study, Mohr et al. showed that female students and first-year students in particular were more comfortable with digital teaching. Students with childcare and job obligations also benefited from this mode of studying [57]. However, for data protection reasons, in our study. Most sites had refrained from collecting socio-demographic data, as from the survey conclusions on the social status of the students may be drawn, so that identifiability of individuals had to be ruled out. Therefore, it is not possible for us to say whether the positive evaluation of asynchronous digital teaching in particular is also related to a high proportion of female students or students with childcare / job obligations. At the very least, this cannot be ruled out, since the proportion of female students is between two thirds and three quarters of the medical students at our faculties. Therefore, we recommend further studies to examine the individual needs of different medical student groups in terms of the optimal form of instruction in order to tailor teaching methods to each medical school’s specific student population.

The results of the present study should be considered in light of its limitations. Regarding the study design, individual students could not be identified due to data protection regulations, and no pseudonyms were used. Thus, we are not dealing with completely the same collective Furthermore, since the answers to various questions, especially in conjunction with free-text comments, allow conclusions to be drawn about the students’ social situation (and thus highly sensitive personal information), in the majority of the surveys, at the discretion of the individual sites, these socio-demographic data were not recorded in order to exclude the potential identifiability of individuals. In addition, the findings are based on cross-sectional data collected at two timepoints with an overall short time interval and therefore cannot predict whether the negative effects found for students from the COVID-19 pandemic will have a long-term impact on students’ learning and success. Here, for example, future exam grades will need to show whether the COVID-19 pandemic has a long-term negative impact on our students. Last but not least and due to site specifics, data for some sites is only available for certain semesters of study or only for the second semester under COVID-19 conditions. In addition, the response rate was just over 20 % because participation in the survey was voluntary. This response rate is within the normal range of student response rates in our medical schools. Nevertheless, this means that a self-selection or volunteer bias cannot be ruled out. It seems conceivable that students who already have disadvantages in the use of digital media (for example, due to insufficient technical equipment or poor Internet connection) participated less because of these problems. Therefore, it seems possible that the reported and discussed disadvantages of socially worse off students are even more frequent than was shown in this evaluation. This makes it all the more necessary for medical schools to address the social disadvantages of students so that they are not left behind in digital teaching. Students who work or have to care for children may also have participated less frequently due to time constraints. Also, considering the study of Mohr et al. [57], the preference for (asynchronous and therefore more flexible) digital teaching offers could possibly be underestimated.

An open research question relates to medical students’ perceptions of examinations during the COVID-19 pandemic. Investigating students’ preferences for teaching design in post–COVID-19 times, which should be anchored in the curriculum, is another interesting research topic. What should be the “new normal” in medical training after the pandemic? Which of the advantages observed by the students should find their way into medical teaching on a permanent basis and which prerequisites still have to be fulfilled for this? It is therefore very important to clarify which competencies medical teachers need in order to be prepared for the future. In addition, the needs of medical faculties, for example in terms of technological infrastructure, should be explored more closely in order to create better conditions for digital teaching and learning and to develop greater adaptability to future requirements.

It appears that the COVID-19 pandemic will be with us for some time to come. In order to determine the most appropriate digital teaching methods for medical education, it is critical to involve students in the shaping of the learning design [58] and to further evaluate student performance on a regular basis [17]. A high degree of digital preparedness in medical education will be required in the future, not only in the event of further health crises [59], but also in adapting to impacts of the climate crisis [60].

## Conclusions

This study presents student perceptions of primarily digital medical education during the first two semesters impacted by the COVID-19 pandemic at Bavarian faculties. Areas for improvement emerged in terms of information dissemination and ensuring access to synchronous online teaching for all students. Several students did not have a sufficiently stable Internet connection to participate in synchronous or asynchronous online teaching, some students did not have sufficient technical equipment. For the future of digital teaching, it is therefore particularly important to provide socially disadvantaged students with technical support so that they are not left behind.

In the future, students would like to see primarily on-site teaching supplemented by teaching recordings. Additional scripts and additional online self-tests were also very important to students. In digital teaching, asynchronous offerings were preferred, most likely because they allow more flexibility. Such asynchronous offerings can probably also very usefully supplement face-to-face teaching in the future, possibly also including the aforementioned teaching recordings, scripts and self-tests. Faculties should take students’ concerns seriously and ensure opportunities to perform practical trainings. Even in times of pandemic, these activities should be maintained as far as possible, as they can hardly be moved into the digital space. The students’ major concern was the lack of social exchange with other students. For the future, faculties must find ways to enable this, even if there is an increased focus on digital teaching. Campus life is not only an integral part of the study time, but also prevents the social isolation of students. This would also increase the level of digital preparedness in medical schools. Future research should address what medical students expect from teaching after COVID-19 and explore the extent to which structural and teaching frameworks need to evolve.

## Supplementary Information

**Additional file 1.**

**Additional file 2.**

**Additional file 3.**

## Data Availability

The datasets generated and analyzed during the current study are not publicly available to prevent comparability of evaluation data between individual study sites. The full aggregated dataset can be found in the supplement. Free text comments cannot be provided for data protection reasons. Should data from the dataset be required, Dr. Christopher Holzmann-Littig can be contacted at christopher.holzmann-littig@mri.tum.de.

## Abbreviations

COVID-19: Coronavirus SARS-CoV-2
BEME: Best Evidence Medical Education
CHE: Centrum für Hochschulentwicklung
DZHW: Deutsches Zentrum für Hochschul- und Wissenschaftsforschung
HIS: Institut für Hochschulentwicklung
KMB: Kompetenznetz Medizinlehre Bayern
UniA: Universität Augsburg
FAU: Friedrich-Alexander-Universität Erlangen-Nürnberg
LMU: Ludwig-Maximilians-Universität München
TUM: Technische Universität München
UR: Universität Regensburg
JMU: Julius-Maximilians-Universität Würzburg
*n*: sample size
*SD*: standard deviation
*p*: *p*-value
*M*: mean value

## Footnotes

1 At FAU there are two human medicine courses of study: Medicine and Medicine Erlangen-Nürnberg/Bayreuth. The latter is a degree program with clinical education at the Medical Campus Upper Franconia in cooperation with the Campus Hospital Bayreuth. Both courses of study were surveyed.

2 only asked at LMU and TUM
