# Supplementary material for: One academic year under COVID-19 conditions: two multicenter cross-sectional evaluation studies among medical students in Bavarian medical schools, Germany students’ needs, difficulties, and concerns about digital teaching and learning
Source: BMC Med Educ. 2022 Jun 10;22:450. doi: 10.1186/s12909-022-03480-x (PMC9183753; doi:10.1186/s12909-022-03480-x)
Supplement: Supplementary file 1 — Additional file 1. [file 12909_2022_3480_MOESM1_ESM.docx]

Supplement 1 [questionnare]

**German version (original)**

**Organisation**

Ich wusste, wo ich Informationen zum Modul- und/oder Semesterablauf finde. *[5-stufige Likert-Skala]*

- trifft voll zu - trifft gar nicht zu
- k.A.

Die zur Verfügung gestellten Informationen waren hilfreich. *[5-stufige Likert-Skala]*

- trifft voll zu - trifft gar nicht zu
- k.A.

Ich wusste, an wen ich mich bei Fragen zum Modul- und/oder Semesterablauf wenden kann. *[5-stufige Likert-Skala]*

- trifft voll zu - trifft gar nicht zu
- k.A.

Allgemeine Kommentare zur Organisation: *[Freitext]*

**Technik**

Mit welchen Endgeräten haben Sie überwiegend an der digitalen Lehre teilgenommen? *[Mehrfachauswahl]*

- Desktop PC
- Laptop
- Tablet
- Smartphone

Mit meiner technischen Ausstattung kann ich an den Online-Präsenzveranstaltungen teilnehmen. *[Einfachauswahl]*

- Ja
- Teilweise
- Nein

Ich kann eine Webcam für Online-Präsenzseminare nutzen. *[Einfachauswahl]*

- Ja
- Teilweise
- Nein

Meine Internetverbindung ist stabil genug für interaktive Online-Präsenzveranstaltungen. *[Einfachauswahl]*

- Ja
- Teilweise
- Nein

Meine Internetverbindung ist stabil genug, um Lehraufzeichnungen/Lehrvideos anzusehen. *[Einfachauswahl]*

- Ja
- Teilweise
- Nein

**Kommunikation und Interaktion**

Mir fehlt der persönliche Kontakt zu den Lehrpersonen. *[5-stufige Likert-Skala]*

- trifft voll zu - trifft gar nicht zu
- k.A.

Mir fehlt der persönliche Kontakt zu meinen Kommilitoninnen und Kommilitonen. *[5-stufige Likert-Skala]*

- trifft voll zu - trifft gar nicht zu
- k.A.

Über welche Kommunikationsmittel haben Sie überwiegend Kontakt zu den Lehrenden gehalten? *[Mehrfachauswahl]*

- E-Mail
- Lernplattform
- WhatsApp und ähnliches
- Facebook
- Instagram
- Twitter
- Telefon
- Videokonferenzsysteme
- Präsenz-Treffen
- Sonstiges
  - Und zwar: *[Freitext]*

Über welche Kommunikationsmittel haben Sie überwiegend Kontakt zu Ihren Kommilitoninnen und Kommilitonen gehalten? [Mehrfachauswahl]

- E-Mail
- Lernplattform
- WhatsApp und ähnliches
- Facebook
- Instagram
- Twitter
- Telefon
- Videokonferenzsysteme
- Präsenz-Treffen
- Sonstiges
  - Und zwar: *[Freitext]*

**Online-Lehre**

Welche Befürchtungen hatten Sie zu Semesterbeginn bezüglich eines digitalen Semesters? *[Mehrfachauswahl]*

- Mangelhafte Informationen zur Organisation von Seiten der Fakultät
- Keine ausreichende technische Ausstattung im persönlichen Umfeld
- Keine ausreichenden digitalen Kenntnisse
- Keine ausreichenden Kenntnisse zur Verwendung der Lernplattform
- Fehlende Möglichkeit, bei Unklarheiten die Lehrperson sofort zu fragen
- Fehlende Möglichkeit, praktische Übungen durchzuführen
- Zu wenige Lehrinhalte, die online gut zu vermitteln sind
- Mangelnder sozialer Austausch mit den Lehrenden
- Mangelnder sozialer Austausch mit meinen Kommilitoninnen und Kommilitonen
- Fehlende Trennung zwischen Lernen und Freizeit
- Sonstiges
  - Und zwar: *[Freitext]*

Zu welcher Tageszeit haben Sie die Online-Lehrmaterialien am meisten bearbeitet/genutzt? *[Einfachauswahl]*

- Morgens
- Vormittags
- Mittags
- Nachmittags
- Abends
- über den Tag verteilt

Generell war das aktuelle Semester deutlich anstrengender als das letzte. *[5-stufige Likert-Skala]*

- trifft voll zu - trifft gar nicht zu
  - Gegebenenfalls: Warum war das aktuelle Semester für Sie anstrengender als das vorherige Semester? *[Freitext]*
- k.A.

Das Bearbeiten der Lerneinheiten fällt mir durch die flexible Zeiteinteilung: *[5-stufige Likert-Skala]*

- viel leichter – viel schwerer
- k.A.

**Gesamtbeurteilung**

Insgesamt beurteile ich das digitale Semester mit: *[5-stufige Likert-Skala]*

- sehr gut – mangelhaft
- k.A.

Welche Lehrform gefällt Ihnen am besten? Bitte priorisieren Sie die folgenden Lehrformen in absteigender Reihenfolge. *[Anordnung per Drag & Drop]*

- Präsenzunterricht
- Online-Lehre ohne synchronen Unterricht, z.B. Podcasts, Videoaufzeichnungen, Lehrfilme, Online-Lernfälle, Folien
- Online-Lehre mit synchronen Elementen, z.B. Zoom-Konferenzen, Online-Quizz, Chat

Ich wünsche mir, dass die klassischen Präsenztermine in den Lehrveranstaltungen in Zukunft verstärkt durch folgende Online-Aktivitäten ergänzt werden: *[Mehrfachauswahl]*

- Live-Unterricht/Webinar (z.B. über Zoom oder Adobe Connect)
- Online-Kommunikation (z.B. über Moodle)
- Videoaufzeichnung/Lehraufzeichnung
- Lehrvideo/Web-Tutorial
- Podcast (Audio-Aufzeichnung)
- Skript u.ä. (z.B. Foliensatz, Zusammenfassung, Zeitschriftenartikel)
- Online-(Gruppen-)Aufgabe
- Online-Lernfall
- Selbstlerntest (z.B. MC-Fragen, AMBOSS-Fragesitzung)
- Sonstige
  - Und zwar: *[Freitext]*

Welche Befürchtungen haben Sie aufgrund Ihrer aktuellen Erfahrungen im digitalen Semester bezüglich des kommenden Semesters? *[Mehrfachauswahl]*

- Mangelhafte Informationen zur Organisation von Seiten der Fakultät
- Unzureichende Verzahnung von Präsenz- und digitaler Lehre
- Keine ausreichende technische Ausstattung im persönlichen Umfeld
- Keine ausreichenden digitalen Kenntnisse
- Keine ausreichenden Kenntnisse zur Verwendung der Lernplattform
- Fehlende Möglichkeit, bei Unklarheiten die Lehrperson sofort zu fragen
- Fehlende Möglichkeit, praktische Übungen durchzuführen
- Zu wenige Lehrinhalte, die online gut zu vermitteln sind
- Mangelnder sozialer Austausch mit den Lehrenden
- Mangelnder sozialer Austausch mit meinen Kommilitoninnen und Kommilitonen
- Fehlende Trennung zwischen Lernen und Freizeit
- Sonstiges
  - Und zwar: *[Freitext]*

Folgendes war am digitalen Semester besonders gelungen und sollte beibehalten werden: *[Freitext]*

Folgendes sollte an zukünftigen digitalen Lehreinheiten unbedingt verbessert werden: *[Freitext]*

**English version (translation)**

**Organizational framework**

I knew where to find information about the module and/or semester schedule. *[5-point Likert scale]*

- fully applies - does not apply at all
- n/a

The information provided was helpful. *[5-point Likert scale]*

- fully applies - does not apply at all
- n/a

I knew whom to contact if I had any questions about the module and/or semester schedule. *[5-point Likert scale]*

- fully applies - does not apply at all
- n/a

General comments on the organizational framework: *[free text]*

**Technological framework**

Which devices did you predominantly use to participate in digital teaching? *[multiple choice]*

- Desktop PC
- Laptop
- Tablet
- Smartphone

With my technical equipment, I can participate in synchronous online sessions. *[single choice]*

- Yes
- Partially
- No

I have a webcam available for synchronous online sessions. *[single choice]*

- Yes
- Partially
- No

My Internet connection is stable enough for interactive, synchronous online sessions. *[single choice]*

- Yes
- Partially
- No

My internet connection is stable enough to watch teaching recordings/instructional videos. *[single choice]*

- Yes
- Partially
- No

**Communication and interaction**

I miss the personal contact with the teachers. *[5-point Likert scale]*

- fully applies - does not apply at all
- n/a

I miss the personal contact with my fellow students. *[5-point Likert scale]*

- fully applies - does not apply at all
- n/a

Through which means of communication have you predominantly maintained contact with the teachers? *[multiple choice]*

- E-mail
- Learning platform
- WhatsApp and similar
- Facebook
- Instagram
- Twitter
- Phone
- Video conferencing systems
- On-site meetings
- Other
  - Namely: *[free text]*

Through which means of communication have you predominantly maintained contact with your fellow students? *[multiple choice]*

- E-mail
- Learning platform
- WhatsApp and similar
- Facebook
- Instagram
- Twitter
- Phone
- Video conferencing systems
- On-site meetings
- Other
  - Namely: *[free text]*

**Online teaching**

Which concerns did you have regarding a digital semester at the beginning of the semester? *[multiple choice]*

- Poor information about the organization on the part of the faculty
- No sufficient technical equipment in the personal environment
- No sufficient digital knowledge
- No sufficient knowledge to use the learning platform
- Lack of possibility to ask the teacher immediately in case of uncertainties
- Lack of possibility to perform practical trainings
- Too little teaching content that can be taught well online
- Lack of social exchange with the teachers
- Lack of social exchange with fellow students
- Lack of separation between learning and leisure
- Other
  - Namely: *[free text]*

At what time of day did you work on / use the online learning materials the most? *[single choice]*

- Morning
- Forenoon
- Noon
- Afternoon
- Evening
- Spread throughout the day

In general, the current semester was much more exhausting than the previous one. *[5-point Likert scale]*

- fully applies - does not apply at all
  - If applicable: Why was the current semester more exhausting for you than the previous semester? *[free text]*
- n/a

The flexible time management makes working on the learning units: *[5-point Likert scale]*

- much easier - much more difficult
- n/a

**Overall assessment**

Overall, I rate the digital semester with: *[5-point Likert scale]*

- very good - poor
- n/a

Which teaching form do you like best? Please prioritize the following teaching forms in descending order. *[arrangement via drag and drop]*

- On-site teaching
- Online teaching without synchronous elements, e.g. podcasts, video recordings, instructional films, online learning cases, slides
- Online teaching with synchronous elements, e.g. Zoom conferences, online quizzes, chat

In the future, I would like to see more of the following online activities supplementing the traditional on-site teaching sessions: *[multiple choice]*

- Synchronous online lesson/webinar (e.g. via Zoom or Adobe Connect)
- Online communication (e.g. via Moodle)
- Teaching recording
- Instructional video/web tutorial
- Podcast (audio recording)
- Script, etc. (e.g. slides, summary, journal article)
- Online (group) task
- Online case-based learning
- Online self-test (e.g. MC questions, AMBOSS question session)
- Other
  - Namely: *[free text]*

Based on your experience in the current digital semester, what are your concerns about the upcoming semester? *[multiple choice]*

- Poor information about the organization on the part of the faculty
- Insufficient integration of on-site and digital teaching
- No sufficient technical equipment in the personal environment
- No sufficient digital knowledge
- No sufficient knowledge to use the learning platform
- Lack of possibility to ask the teacher immediately in case of uncertainties
- Lack of possibility to perform practical trainings
- Too little teaching content that can be taught well online
- Lack of social exchange with the teachers
- Lack of social exchange with fellow students
- Lack of separation between learning and leisure
- Other
  - Namely: *[free text]*

The following was particularly successful about the digital semester and should be maintained: *[free text]*

The following should definitely be improved in future digital teaching units: *[free text]*
