# Supplementary material for: One academic year under COVID-19 conditions: two multicenter cross-sectional evaluation studies among medical students in Bavarian medical schools, Germany students’ needs, difficulties, and concerns about digital teaching and learning
Source: BMC Med Educ. 2022 Jun 10;22:450. doi: 10.1186/s12909-022-03480-x (PMC9183753; doi:10.1186/s12909-022-03480-x)
Supplement: Supplementary file 2 — Additional file 2. [file 12909_2022_3480_MOESM2_ESM.docx]

Supplement 2 [CHERRIES checklist](1)

| **Checklist for Reporting Results of Internet E-Surveys (CHERRIES)** | |
| --- | --- |
| ***Item Category***/***Checklist Item*** | ***Explanation*** |
| **Design** | |
| Describe survey design | Target population: Medical students from Bavarian faculties. De facto closed online survey. |
| **IRB (Institutional Review Board) approval and informed consent process** | |
| IRB approval | All ethics committees approved the conduct of the study or waived further review. |
| Informed consent | Participants gave informed consent by filling in the voluntary survey/clicking on a checkbox. The information on the survey was given according to each faculty’s local regulations. Free text comments were erased for data protection reasons before building the complete dataset at TUM. |
| Data protection | Data is stored at each medical school and a Moodle course at UR (both password-protected). No identifying personal data was collected. Free text comments were not shared with other medical schools or names/title designations were blackened before sharing. Datasets are anonymous. No pseudonyms were used. |
| **Development and pre-testing** | |
| Development and testing | The question set had been developed by experts from the Competence Network Medical Education Bavaria. Each medical school was allowed to add additional questions according to local needs. The overall question set was tested by colleagues from the Competence Network Medical Education Bavaria. Each medical school tested the usability/functionality of their own survey. |
| **Recruitment process and description of the sample having access to the questionnaire** | |
| Open survey versus closed survey | De facto closed survey: Either a password was sent out to students of the respective medical school or individual passwords/links were sent to students, according to the practice of the faculty. |
| Contact mode | All participants were invited via E-mail. |
| Advertising the survey | No further advertisement was performed. |
| **Survey administration** | |
| Web/E-mail | The survey was not posted on any website, the link was only sent out via E-mail. The responses were captured automatically. |
| Context | The online survey was conducted using *EvaSys* (*evasys GmbH*): <https://evasys.de> |
| Mandatory/voluntary | Voluntary |
| Incentives | No incentives |
| Time/Date | July 2020 – October 2020 and January 2021 – April 2021 |
| Randomization of items or questionnaires | Items were not randomized. |
| Adaptive questioning | When the answer option "other" was clicked, users were given the opportunity to give a free text comment. If participants indicated that they found the current semester to be much more stressful than the previous semester, they were asked why this was the case (free text question), depending on the respective medical school. |
| Number of items | Basic question set: 29 questions, minor differences due to extensions at all medical schools |
| Number of screens (pages) | Some schools presented the questionnaire on one scroll-down page. If screens were applied, there were approximately 8 of them. |
| Completeness check | Completeness of question sets was not checked during the survey but during analysis; absolute answer frequencies are given in the text. |
| Review step | Reviews were possible before terminating the survey. |
| **Response rates** | |
| Unique site visitor | No IP address checks or cookies were used. |
| View rate | No view rates were recorded. |
| Participation rate | 22.2% in summer semester and 20.9% in winter semester |
| Completion rate | To submit the survey, participants had to go through the survey until they reached the finish button. In each question the option not to answer was given without need for early termination of the survey. Absolute numbers of participants answering a question are given in the text. |
| **Preventing multiple entries from the same individual** | |
| Cookies used | No cookies were used. |
| IP check | No IP address checks were performed for data protection reasons. |
| Log file analysis | No log file analysis was performed for data protection reasons. |
| Registration | Users had to click on the individualized link or fill in the personal/medical school-wide password, depending on the respective medical school. |
| **Analysis** | |
| Handling of incomplete questionnaires | Incomplete questionnaires were analyzed, absolute numbers of participants answering the respective question are given in the text. |
| Questionnaires submitted with an atypical timestamp | No analyses for atypical time stamps were performed. |
| Statistical correction | No weighting of items was performed. |

1. Eysenbach G. Improving the quality of Web surveys: the Checklist for Reporting Results of Internet E-Surveys (CHERRIES). J Med Internet Res. 2004;6(3):e34-e.
