# Supplementary material for: One academic year under COVID-19 conditions: two multicenter cross-sectional evaluation studies among medical students in Bavarian medical schools, Germany students’ needs, difficulties, and concerns about digital teaching and learning
Source: BMC Med Educ. 2022 Jun 10;22:450. doi: 10.1186/s12909-022-03480-x (PMC9183753; doi:10.1186/s12909-022-03480-x)
Supplement: Supplementary file 3 — Additional file 3. [file 12909_2022_3480_MOESM3_ESM.docx]

Supplement 3 [quantitative results]

| **Answer options** | **Results summer semester 2020**  ***n*=1565** | **Results winter semester 2020/21**  ***n*=1727** | ***p*** |
| --- | --- | --- | --- |
| **Organizational framework** | | | |
| I knew where to find information about the module and/or semester schedule. *[5-point Likert scale]; M (SD)* | | | |
| - fully applies - does not apply at all | 3.7 (1.1) | 4.0 (1.0) | <0.001 |
| - n/a | 65 | 32 |  |
| The information provided was helpful. *[5-point Likert scale]; M (SD)* | | | |
| - fully applies - does not apply at all | 3.8 (0.9) | 3.9 (0.9) | <0.001 |
| - n/a | 69 | 38 |  |
| I knew whom to contact if I had any questions about the module and/or semester schedule. *[5-point Likert scale]; M (SD)* | | | |
| - fully applies - does not apply at all | 3.6 (1.2) | 3.8 (1.2) | <0.001 |
| - n/a | 80 | 53 |  |
| General comments on the organizational framework: *[free text]* | | | |
| Technological framework | | | |
| Which devices did you predominantly use to participate in digital teaching? *[multiple choice]; n (percent)* | | | |
| - Desktop PC | 162 (10.6%) | 217 (12.6%) | 0.086 |
| - Laptop | 1303 (85.5%) | 1492 (86.4%) | 0.464 |
| - Tablet | 643 (42.2%) | 712 (41.2%) | 0.578 |
| - Smartphone | 241 (15.8%) | 192 (11.1%) | <0.001 |
| - n/a | 41 | 0 |  |
| With my technical equipment, I can participate in synchronous online sessions. *[single choice]; n (percent)* | | | |
| - Yes | 1389 (92.5%) | 1594 (93.7%) | 0.153 |
| - Partially | 107 (7.1%) | 105 (6.2%) |  |
| - No | 6 (0.4%) | 2 (0.1%) |  |
| - n/a | 63 | 26 |  |
| I have a webcam available for synchronous online sessions. *[single choice]; n (percent)* | | | |
| - Yes | 1361 (90.7%) | 1570 (92.7%) | 0.002 |
| - Partially | 96 (6.4%) | 104 (6.1%) |  |
| - No | 43 (2.9%) | 19 (1.1%) |  |
| - n/a | 65 | 34 |  |
| My internet connection is stable enough for interactive, synchronous online sessions. *[single choice]; n (percent)* | | | |
| - Yes | 940 (62.6%) | 1030 (60.6%) | 0.469 |
| - Partially | 523 (34.8%) | 623 (36.6%) |  |
| - No | 38 (2.5%) | 48 (2.8%) |  |
| - n/a | 64 | 26 |  |
| My internet connection is stable enough to watch teaching recordings/instructional videos. *[single choice]; n (percent)* | | | |
| - Yes | 1247 (83.0%) | 1419 (83.5%) | 0.746 |
| - Partially | 243 (16.2%) | 264 (15.5%) |  |
| - No | 12 (0.8%) | 17 (1.0%) |  |
| - n/a | 63 | 27 |  |
| Communication and interaction | | | |
| I miss the personal contact with the teachers. *[5-point Likert scale]; M (SD)* | | | |
| - fully applies - does not apply at all | 3.6 (1.2) | 3.8 (1.2) | <0.001 |
| - n/a | 26 | 37 |  |
| I miss the personal contact with my fellow students. *[5-point Likert scale]; M (SD)* | | | |
| - fully applies - does not apply at all | 4.4 (1.0) | 4.6 (0.9) | <0.001 |
| - n/a | 22 | 35 |  |
| Through which means of communication have you predominantly maintained contact with the teachers? *[multiple choice]; n (percent)* | | | |
| - E-mail | 1187 (77.9%) | 1351 (78.2%) | 0.815 |
| - Learning platform | 975 (64.0%) | 962 (55.7%) | <0.001 |
| - WhatsApp and similar | 36 (2.4%) | 30 (1.7%) | 0.207 |
| - Facebook | 3 (0.2%) | 3 (0.2%) | 0.878 |
| - Instagram | 1 (0.1%) | 2 (0.1%) | 0.638 |
| - Twitter | 1.0 (0.1%) | 0 (0.0%) | 0.287 |
| - Phone | 49 (3.2%) | 34 (2.0%) | 0.025 |
| - Video conferencing systems | 948 (62.2%) | 960 (55.6%) | <0.001 |
| - On-site meetings | 90 (6.7%) | 185 (12.0%) | <0.001 |
| - Other | 11 (0.7%) | 11 (0.6%) | 0.768 |
| - Namely: *[free text]* |  | |  |
| - n/a | 41 | 0 |  |
| Through which means of communication have you predominantly maintained contact with your fellow students? *[multiple choice]; n (percent)* | | | |
| - E-mail | 217 (14.2%) | 182 (10.5%) | 0.001 |
| - Learning platform | 123 (8.1%) | 107 (6.2%) | 0.037 |
| - WhatsApp and similar | 1464 (96.1%) | 1640 (95.0%) | 0.132 |
| - Facebook | 376 (24.7%) | 300 (17.4%) | <0.001 |
| - Instagram | 356 (23.4%) | 433 (25.1%) | 0.256 |
| - Twitter | 6 (0.4%) | 9 (0.5%) | 0.593 |
| - Phone | 643 (42.2%) | 682 (39.5%) | 0.118 |
| - Video conferencing systems | 572 (37.5%) | 789 (45.7%) | <0.001 |
| - On-site meetings | 699 (52.2%) | 676 (43.9%) | <0.001 |
| - Other | 38 (2.5%) | 38 (2.2%) | 0.581 |
| - Namely: *[free text]* |  | |  |
| - n/a | 41 | 0 |  |
| Online teaching | | | |
| Which concerns did you have regarding a digital semester at the beginning of the semester? *[multiple choice]; n (percent)* | | | |
| - Poor information about the organization on the part of the faculty | 1114 (71.2%) | 956 (55.4%) | <0.001 |
| - No sufficient technical equipment in the personal environment | 196 (12.5%) | 163 (9.4%) | 0.005 |
| - No sufficient digital knowledge | 201 (13.2%) | 180 (10.4%) | 0.014 |
| - No sufficient knowledge to use the learning platform | 236 (15.1%) | 192 (11.1%) | <0.001 |
| - Lack of possibility to ask the teacher immediately in case of uncertainties | 652 (41.7%) | 716 (41.5%) | 0.906 |
| - Lack of possibility to perform practical trainings | 1233 (78.8%) | 1382 (80.0%) | 0.380 |
| - Too little teaching content that can be taught well online | 830 (53.0%) | 824 (47.7%) | 0.002 |
| - Lack of social exchange with the teachers | 710 (45.4%) | 923 (53.4%) | <0.001 |
| - Lack of social exchange with fellow students | 1224 (78.3%) | 1456 (84.3%) | <0.001 |
| - Lack of separation between learning and leisure | 848 (54.2%) | 1059 (61.3%) | <0.001 |
| - Other | 46 (3.0%) | 43 (2.5%) | 0.375 |
| - Namely: *[free text]* |  | |  |
| - n/a | 41 | 0 |  |
| At what time of day did you work on / use the online learning materials the most? *[single choice]; n (percent)* | | | |
| - Morning | 156 (10.4%) | 178 (10.5%) | 0.122 |
| - Forenoon | 358 (23.9%) | 408 (24.1%) |  |
| - Noon | 39 (2.6%) | 48 (2.8%) |  |
| - Afternoon | 89 (5.9%) | 142 (8.4%) |  |
| - Evening | 106 (7.1%) | 128 (7.5%) |  |
| - Spread throughout the day | 752 (50.1%) | 792 (46.7%) |  |
| - n/a | 65 | 31 |  |
| In general, the current semester was much more exhausting than the previous one. *[5-point Likert scale]; M (SD)* | | | |
| - fully applies - does not apply at all | 3.1 (1.4) | 3.1 (1.4) | 0.675 |
| - - If applicable: Why was the current semester more exhausting for you than the previous semester? *[free text]* |  | |  |
| - n/a | 157 | 283 |  |
| The flexible time management makes working on the learning units: *[5-point Likert scale]; M (SD)* | | | |
| - much easier - much more difficult | 3.7 (1.2) | 3.6 (1.1) | 0.009 |
| - n/a | 97 | 92 |  |
| Overall assessment | | | |
| Overall, I rate the digital semester with: *[5-point Likert scale]; M (SD)* | | | |
| - very good - poor | 2.5 (1.1) | 2.5 (1.0) | 0.341 |
| - n/a | 71 | 48 |  |
| Which teaching form do you like best? Please prioritize the following teaching forms in descending order. *[arrangement via drag and drop]; n (percent)* | | | |
| - On-site teaching | First choice  767 (55.5%) | First choice  1031 (65.7%) |  |
|  | Second choice  339 (24.5%) | Second choice  305 (19.4%) |  |
|  | Third choice  264 (19.7%) | Third choice  232 (15.1%) |  |
| - Online teaching without synchronous elements, e.g. podcasts, video recordings, instructional films, online learning cases, slides | First choice  378 (27.4%) | First choice  329 (21.0%) |  |
|  | Second choice  441 (31.9%) | Second choice  488 (31.1%) |  |
|  | Third choice  552 (41.2%) | Third choice  740 (48.1%) |  |
| - Online teaching with synchronous elements, e.g. Zoom conferences, online quizzes, chat | First choice  236 (17.1%) | First choice  209 (13.3%) |  |
|  | Second choice  600 (43.4%) | Second choice  772 (49.2%) |  |
|  | Third choice  525 (39.2%) | Third choice  566 (36.8%) |  |
| - n/a | First choice  184 | First choice  158 |  |
|  | Second choice  184 | Second choice  158 |  |
|  | Third choice  224 | Third choice  189 |  |
| In the future, I would like to see more of the following online activities supplementing the traditional on-site teaching sessions: *[multiple choice]; n (percent)* | | | |
| - Synchronous online lesson/webinar (e.g. via Zoom or Adobe Connect) | 566 (37.1%) | 670 (38.8%) | 0.332 |
| - Online communication (e.g. via Moodle) | 353 (23.2%) | 376 (21.8%) | 0.343 |
| - Teaching recording | 1258 (82.5%) | 1311 (75.9%) | <0.001 |
| - Instructional video/web tutorial | 856 (56.2%) | 854 (49.5%) | <0.001 |
| - Podcast (audio recording) | 521 (34.2%) | 361 (20.9%) | <0.001 |
| - Script, etc. (e.g. slides, summary, journal article) | 1066 (69.9%) | 1168 (67.6%) | 0.155 |
| - Online (group) task | 151 (9.9%) | 173 (10.0%) | 0.917 |
| - Online case-based learning | 497 (32.6%) | 547 (31.7%) | 0.568 |
| - Online self-test (e.g. MC questions, AMBOSS question session) | 899 (59.0%) | 970 (56.2%) | 0.104 |
| - Other | 7 (0.5%) | 16 (1.0%) | 0.121 |
| - Namely: *[free text]* |  |  |  |
| - n/a | 41 | 0 |  |
| Based on your experience in the current digital semester, what are your concerns about the upcoming semester? *[multiple choice]; n (percent)* | | | |
| - Poor information about the organization on the part of the faculty | 588 (37.6%) | 542 (31.4%) | <0.001 |
| - Insufficient integration of on-site and digital teaching | 825 (54.1%) | 791 (45.8%) | <0.001 |
| - No sufficient technical equipment in the personal environment | 68 (4.3%) | 72 (4.2%) | 0.803 |
| - No sufficient digital knowledge | 42 (2.8%) | 47 (2.7%) | 0.955 |
| - No sufficient knowledge to use the learning platform | 47 (3.0%) | 44 (2.5%) | 0.426 |
| - Lack of possibility to ask the teacher immediately in case of uncertainties | 383 (24.5%) | 521 (30.2%) | <0.001 |
| - Lack of possibility to perform practical trainings | 1044 (66.7%) | 1188 (68.8%) | 0.202 |
| - Too little teaching content that can be taught well online | 638 (40.8%) | 713 (41.3%) | 0.763 |
| - Lack of social exchange with the teachers | 604 (38.6%) | 842 (48.8%) | <0.001 |
| - Lack of social exchange with fellow students | 1005 (64.2%) | 1311 (75.9%) | <0.001 |
| - Lack of separation between learning and leisure | 653 (41.7%) | 880 (51.0%) | <0.001 |
| - Other | 57 (3.7%) | 47 (2.7%) | 0.100 |
| - Namely: *[free text]* |  | |  |
| - n/a | 41 | 0 |  |
| The following was particularly successful about the digital semester and should be maintained: *[free text]* | | | |
| The following should definitely be improved in future digital teaching units: *[free text]* | | | |

*Notes*: *n* = number of responses; *p* = p-value; *M* = mean value; *SD* = standard deviation; *n/a* = no answer or not applicable.
